# Impact of Cannabis Use on Overall Brain Volume in first episode psychosis Patients

**DOI:** 10.1192/j.eurpsy.2025.1695

**Published:** 2025-08-26

**Authors:** C. Ovejas-Catalán, D. Tordesillas Gutiérrez, E. Marco de Lucas, M. Drake Pérez, P. Fuentes-Pérez, P. Suárez Pinilla, D. de la Sierra Biddle, M. L. Ramírez Bonilla, J. Vázquez-Bourgon, R. Pérez Iglesias

**Affiliations:** 1 Valdecilla Biomedical Research Institute - IDIVAL; 2 Institute of Physics of Cantabria (UC-CSIC), Spanish National Research Council (CSIC); 3 Radiodiagnosis Department, University Hospital Marqués de Valdecilla.; 4 University of Cantabria (UC); 5 Psychiatry Department, University Hospital Marqués de Valdecilla., Santander, Spain

## Abstract

**Introduction:**

Neuroimaging studies show that schizophrenia is linked to reduced grey and white matter volumes and increased cerebrospinal fluid. Cannabis use, a widely known risk factor for psychosis, is associated with poorer clinical outcomes, although the mechanisms underlying this association remain unknown.

**Objectives:**

This study aims to explore the effect of cannabis use on brain volumes in individuals with a first episode of psychosis, comparing users and non-users.

**Methods:**

A cross-sectional study with 207 participants was conducted at the Cantabria Early Psychosis Intervention Program (ITPCan) in Santander, Spain, from January 2020 to July 2024. Clinical, sociodemographic, and cannabis use data were collected. Structural magnetic resonance imaging (sMRI) scans were obtained using a Philips 3.0T MRI machine with T1-weighted sequences. Voxel-based morphometry (VBM) analysis was conducted using the CAT12 toolbox to assess relative volume measures of white matter (WM), gray matter (GM), and cerebrospinal fluid (CSF), accounting for individual differences.Statistical analyses were performed by SPSS 23.0, with a significance of 0.05, including mean comparisons and multivariate analysis of covariance controlling for age, sex, and educational level.

**Results:**

Out of the total sample, 106 patients underwent sMRI, including 44 men and 62 women, with an average age of 36.9 years. In terms of education, 47.2% had achieved basic level, while 52.8% had higher education. Regarding cannabis-related variables, 28 participants (26.5%) were identified as users; the average age of initiation was 17.1 years, with consumption occurring around 6.5 days per week and 6. 7 joints per day.

Non-user group showed slightly higher mean CSF and WM volumes compared to users (CSF=18.65 vs. 17.56; WM=36.49 vs.35.99), but these differences did not reach statistical significance (p= 0.154; p = 0.265). In contrast, cannabis users showed a significantly greater relative mean GM volume (46.37 vs. 45.12, p = 0.037). However, these differences did not reach statistical significance after adjusting for age, sex, and education.

**Conclusions:**

Cannabis use is associated with greater GM volumes among individual with a first episode of psychosis. However, these differences did not remain significant after adjusting for age sex and education. GM differences could largely be attributed to the age disparity between both groups, with cannabis users being significantly younger than non-users (27 vs. 40.8 years).

Further research into the underlying mechanisms and long-term studies are needed to provide a clearer understanding of how cannabis use affects brain structure over time.

**Disclosure of Interest:**

None Declared

